# Neurophysiological mechanisms underlying the differential effect of reward prospect on response selection and inhibition

**DOI:** 10.1038/s41598-023-37524-z

**Published:** 2023-07-05

**Authors:** Anna Helin Koyun, Ann-Kathrin Stock, Christian Beste

**Affiliations:** 1 Cognitive Neurophysiology, Department of Child and Adolescent Psychiatry, Faculty of Medicine, TU Dresden, Schubertstrasse 42, 01309 Dresden, Germany; 2 Faculty of Medicine, University Neuropsychology Center, TU Dresden, Dresden, Germany; 3 Biopsychology, Faculty of Psychology, School of Science, TU Dresden, Dresden, Germany

**Keywords:** Human behaviour, Cognitive neuroscience, Reward

## Abstract

Reward and cognitive control play crucial roles in shaping goal-directed behavior. Yet, the behavioral and neural underpinnings of interactive effects of both processes in driving our actions towards a particular goal have remained rather unclear. Given the importance of inhibitory control, we investigated the effect of reward prospect on the modulatory influence of automatic versus controlled processes during response inhibition. For this, a performance-contingent monetary reward for both correct response selection and response inhibition was added to a Simon NoGo task, which manipulates the relationship of automatic and controlled processes in Go and NoGo trials. A neurophysiological approach was used by combining EEG temporal signal decomposition and source localization methods. Compared to a non-rewarded control group, rewarded participants showed faster response execution, as well as overall lower response selection and inhibition accuracy (shifted speed-accuracy tradeoff). Interestingly, the reward group displayed a larger interference of the interactive effects of automatic versus controlled processes during response inhibition (i.e., a larger Simon NoGo effect), but not during response selection. The reward-specific behavioral effect was mirrored by the P3 amplitude, underlining the importance of stimulus–response association processes in explaining variability in response inhibition performance. The selective reward-induced neurophysiological modulation was associated with lower activation differences in relevant structures spanning the inferior frontal and parietal cortex, as well as higher activation differences in the somatosensory cortex. Taken together, this study highlights relevant neuroanatomical structures underlying selective reward effects on response inhibition and extends previous reports on the possible detrimental effect of reward-triggered performance trade-offs on cognitive control processes.

## Introduction

Everyday, we are confronted with dynamically changing environments. To meet the requirements of these changes, we engage in goal-directed behavior, which is crucially based on the ability to inhibit inappropriate responses^1^. Several factors have been suggested to influence inhibitory processes. These include top-down cognitive control^2^ and the automaticity of pre-potent responses^3^. Additionally, reward was proposed to be an effective modulator of human behavioral performance^4^ and to influence (inhibitory) cognitive control processes^5,6^. Impairments in (inhibitory) cognitive control and reward processing have been reported to be key characteristics of several clinical populations, including individuals with substance use disorders and ADHD^7–9^. It is therefore crucial to better understand how cognitive control and reward prospect interact in driving goal-directed behavior.

When behavioral patterns or response tendencies are conflicting with our goals, cognitive control needs to be exerted to resolve/overcome this conflict by withholding/inhibiting a (pre-potent) response. It has been shown that once (monetary) rewards are at stake, individuals tend to increase the amount of cognitive resources and effort spent towards a certain task/goal^5,10^. A widely accepted postulation is that (monetary) rewards can alter the biological system by generating a “motivated state” that in turn leads to adaptations in behavior and cognitive processing^11–13^. Importantly, higher motivation does not automatically contribute to increased cognitive control^14^. Based on the type of reward manipulation and reward rules, different effects have been reported. Performance-contingent rewards (i.e., reward for fast and accurate responses) were shown to facilitate behavioral optimization^10,15^, whereas performance-noncontingent rewards (e.g., random rewards in a subset of trials) appeared not to modulate cognitive control strategies^16^. Given that performance-contingent rewards increase the ambitious effort engaged in cognitive control^4^, they may facilitate effortful cognitive control processes such as inhibitory control^17,18^, conflict processing performance^10,15^ and conflict adaptation (i.e., an increase in effort/processing capacities when conflict is experienced)^19^. Furthermore, performance-contingent rewards have been suggested to lead to behavioral stability by facilitating the maintenance of context information^20,21^. Considering the underlying neural mechanisms, dopamine (DA) has been suggested to be a crucial element in mediating the interactive effects of motivation (e.g., through monetary reward) and cognitive control, including inhibition^22,23^. Prefrontal DA is generally assumed to facilitate relevant task/goal representations (e.g., via tonic DA release), and cortical DA has recently been suggested to facilitate the disengagement from automatic response tendencies (regardless of whether this facilitates or impedes behavior)^24^. Moreover, phasic striatal DA signalling has been suggested to be crucial for optimizing the allocation of cognitive efforts to obtain a reward^23,25^.

While reward prospect and the associated DA modulations are often reported to facilitate (controlled) behavior, automaticity (i.e., probability of executing a pre-potent response) has been shown to increase the need for cognitive control and obstruct inhibitory performance^3,26^. Thus, one needs to engage in controlled/instrumental responses to decrease the impact of automatic responses on response inhibition. Experiments combining a “Simon Task” with a “Go/NoGo Task” have shown conjoint effects of automatic and controlled processes during (motor) response inhibition^26,27^. In a Simon Task, response conflicts arise because of congruent (same-side/non-conflicting) or incongruent (opposite-side/conflicting) stimulus–response hand relationships. Response execution is commonly observed to be slower and more prone to errors in incongruent (conflicting) Go trials, whereas faster and more accurate responses have been observed in congruent Go trials^26,28,29^. The difference in accuracy between congruent and incongruent trials yields the Simon effect. According to the dual process account^30^, this is the result of conflicts that arise between “automatic” and “controlled” processing routes. Accordingly, the “direct-route” generates rather automatic response tendencies based on the task-irrelevant stimulus location, which are sufficient to produce correct response executions on congruent trials. During incongruent trials, however, there is a necessity to control these automatic response tendencies, as they would otherwise lead to incorrect responses. This is accomplished via the “indirect route”, which refers to a rather controlled selection of relevant stimulus features indicating the correct response. In the Simon NoGo Task, response inhibition (NoGo) trials are included in a Simon task, thus generating congruent and incongruent NoGo trials. The usually observed pattern of the Simon effect has been shown to reverse when motor response inhibition is required^26^: In congruent NoGo trials, the level of automatic response tendencies is high^3^, thereby leading to increased false alarm rates. In contrast, the impact/ inhibitory control of the “indirect route” reduces automatic response tendencies during incongruent NoGo trials and by that facilitates motor response inhibition, leading to lower false alarm rates^26,27^. We used this Simon NoGo task to investigate if and how performance-contingent reward, and possibly associated DA modulations, alter the degree to which interactive effects of automatic and controlled response selection processes influence inhibitory control, including response inhibition. Given that rewards can impair performance when only one of various response modes/conditions is rewarded (e.g., response execution over inhibition^10^;), we decided to reward both correct response selection and inhibition in the current study (see “Methods” section for reward rule details).

Reward and cognitive control have both been suggested to enhance task-relevant associations, but the underlying neurophysiological mechanisms have remained rather elusive. In the current study, we set out to investigate the interactive effects of reward prospect and cognitive control processes on response inhibition using a neurophysiological approach combining EEG temporal signal decomposition and source localization methods. We were specifically interested in reward prospect-based differences in the magnitude of the interactive effects of automatic and controlled response selection processes modulating motor response inhibition. Previous studies have shown that classical event-related potentials (ERPs) reflect a mixture of perceptual, stimulus-related processes (termed “stimulus codes”) and motor response selection processes (termed “response selection codes”)^27,31^, which co-exist during response inhibition^31^. As a consequence, regular ERPs may only produce precise insights into neurophysiological processes if the intra-individual variability is very low^32,33^. However, evidence suggests that the effects of (monetary) rewards on inhibitory processes are rather heterogeneous^34^, possibly due to differences at the neurophysiological level. Thus, correlates of group-dependent behavioral modulations were not expected to be well-explained by standard averaged ERP waveforms due to the expected trial-to-trial latency variability. In the current study, we therefore applied residue iteration decomposition (RIDE^33^) to overcome this problem and dissociate stimulus codes and inhibitory control codes in the EEG signal. Specifically, RIDE temporally decomposes the EEG signal into several separate component clusters (S-, R-, & C-cluster), with variable intercomponent delays and distinct functional relevance^35^. The S-cluster reflects stimulus-related processes (i.e., perception and attention), the R-cluster refers to response-related processes (i.e., motor response execution), and the C-cluster reflects intermediate processes between the S-, and the R-cluster (i.e., response selection). Each cluster usually comprises different (aspects of) ERP components. Typically, the N1 and P2 components are most strongly shown in the S-cluster, and the P3 component is often best captured by the information in the C- and R-clusters. The N2 ERP has been described to represent both stimulus and response-related processes^36,37^ and can therefore often be found in all three RIDE clusters.

Concerning the expected results, we hypothesized that the performance-contingent reward might improve both response selection and response inhibition (in terms of accuracy). Since reward prospect has been repeatedly shown to shorten reaction times^38–40^, it could however also be possible that responses become faster and accuracy performance might suffer as a result of this. Another potential consequence of such response speeding could be larger accuracy-based Simon effects (i.e., interactive effects of automatic and controlled processes) in the reward context^41^. The interplay of interference inhibition (i.e., the inhibition of interfering effects of distracting stimuli and stimulus properties onto response selection, which is typically most required in incongruent trials) and motor response inhibition (i.e., the inhibition of prepotent motor responses) during NoGo trials has been reported to be modulated by stimulus–response (S–R) translation processes as reflected in the C-cluster^26^, and here, mainly by the NoGo-P3. Given the importance of S–R translation/selection processes for successful response inhibition, we expected the effect of reward prospect itself to be reflected in C-cluster (NoGo-)P3 modulations that reflect/parallel the task effects found on the behavioral level.

Recently, it was further suggested that early attentional and specifically early cognitive resource allocation play a key role in the interaction of automatic and cognitive control processes during response inhibition^42^. This is in accordance with accounts underlining the importance of early ERP components in determining successful response inhibition^43,44^. We therefore anticipated variations in the extent to which modulations in the S-cluster explain observable differences in behavioral performance (especially the interaction between controlled and automatic processes) between the reward intervention and control group. Subsequent source localization analyses were conducted to pinpoint which functional neuroanatomical structures are associated with any reward prospect-based differences in neural activity.

## Material and methods

### Participants

Thirty-eight young and healthy adults [17 males; mean age = 23.89 years; SEM = 0.557] were recruited to participate in the current reward intervention study. The control group comprised another thirty-eight healthy adults [17 males; mean age = 23.92 years; SEM = 0.145], who were randomly drawn from previous applications of this task in our research group. All participants included in the study were between 19 and 33 years old, reported to be healthy (no history of chronic or acute neurological, psychiatric, or somatic disease), and had normal or corrected-to-normal vision. After purpose and procedure of the study had been fully explained, written informed consent was obtained from all participants. Upon completion of the study, all participants received 20 € as monetary compensation. Participants in the reward group additionally received a cumulative monetary reward of up to 6€ for good performance (for further details, please refer to the task description). 

### Task and procedure

**Figure 1:**
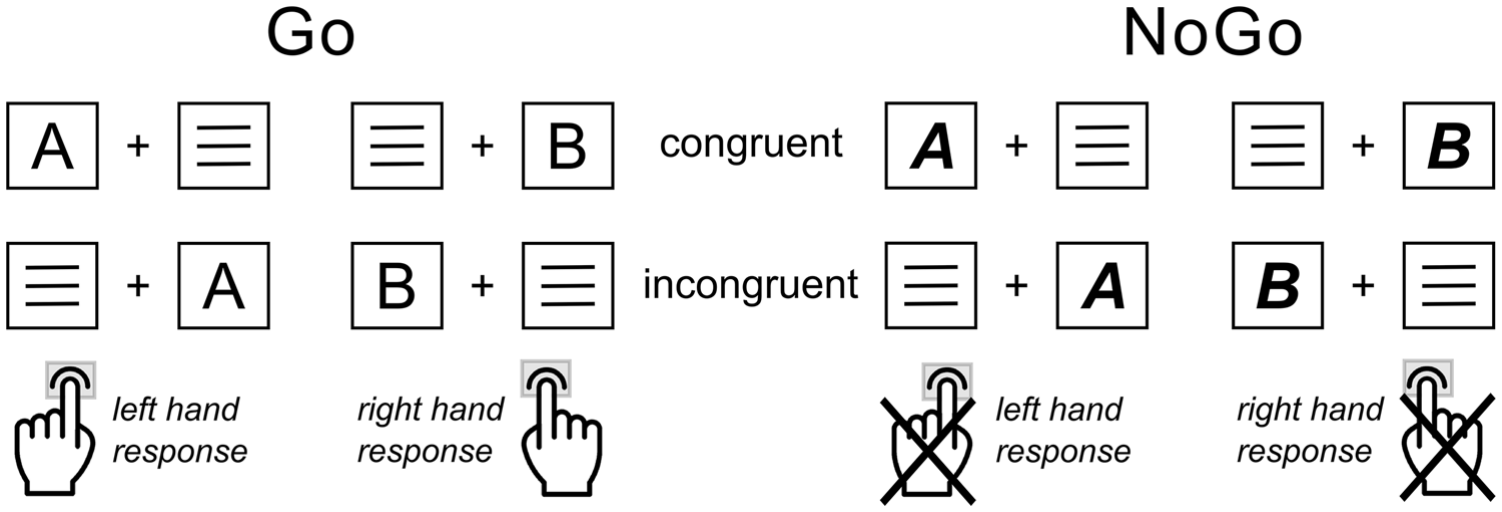
Experimental paradigm: Simon NoGo Task. Illustrated are the possible stimulus–response combinations for congruent/incongruent Go condition (left) and congruent/incongruent NoGo condition (right). Congruent versus incongruent refers to the stimulus–response hand mapping of Go trials. Therefore, congruent trials required a response execution on the side the target letter stimulus was presented on in case of Go trials, whereas the stimulus presentation side does not match up with the stimulus–response hand in incongruent trials.

In the current study, we used a combined Simon and Go/NoGo paradigm^26^, that allows to investigate the influence of automatic versus controlled response tendencies on response inhibition. The experiment was programmed and run using Presentation (Version 22.1, Neurobehavioral Systems Inc., CA, United States). Task design and structure are illustrated in Fig. 1.

The experiment was conducted in a dimly lit, sound-isolated room. For the task, participants were seated at a distance of approximately 60 cm in front of a 24-inch LCD monitor (60 Hz frame rate), on which visual stimuli were presented on black background. During the experiment, a central white fixation cross, accompanied by two lateralized empty white frame boxes (representing possible stimulus positions at about 1.1°–1.7° visual angle from the center), was continuously displayed on the screen. Each trial started with the simultaneous presentation of a single letter target stimulus (i.e., yellow “A” or “B”) and a distractor stimulus (three horizontal white lines; matching the letter stimulus in size) within the two white frame boxes for 200 ms (both sized approx. 0.6° visual angle in height and width). Crucially, the font of the letter stimulus indicated whether the current trial was a Go trial (normal font) or a NoGo trial (***bold-italic*** font). In response to letter stimulus “A” in normal font, participants were instructed to press the left control button with their left index finger. In response to letter “B” in normal font, participants had to press the right control button with their right index finger. This rule applied irrespective of the side (left or right-hand side) that the letter stimulus was presented on. In Go trials, a speed-up sign (i.e., “Schneller!” / “Faster!”) was presented above the fixation cross in case no response was given within 500 ms after stimulus presentation. When the letter stimulus was presented in ***bold-italic*** font (i.e., yellow “***A***” or “***B***”), participants had to withhold associated responses (NoGo trials). Trials in which the letter stimulus was presented in the spatial location matching the associated response hand (i.e., “A” or “***A***” presented on the left-hand side, “B” or “***B***” presented on the right-hand side) were coded as congruent trials. When the letter stimulus was presented at the spatially opposite location with respect to the associated response hand (i.e., “A” or “***A***” presented on the right, “B” or “***B***” presented on the left), the trials were coded as incongruent trials. This creates the following four conditions: (1) congruent Go trials, (2) incongruent Go trials, (3) congruent NoGo trials, and (4) incongruent NoGo trials. Responses to Go condition trials within 1700 ms after stimulus presentation were coded as “correct” or “incorrect”. Furthermore, trials in which no response was obtained until 1700 ms post-stimulus presentation were, dependent on the condition, coded either as Go trial “misses” or NoGo trial “correct omissions”. In the NoGo trials, any response obtained within 1700 ms after stimulus presentation was coded as a “false alarm”. The inter-trial interval (ITI) was jittered between 1300 and 1700 ms. Overall, the experimental paradigm comprised 6 blocks with 120 trials each, resulting in a total number of 720 trials. 70% (504 trials in total, 84 trials per block) of the trials were Go-trials, and 30% (216 trials in total, 36 trials per block) were NoGo trials. In both the Go and NoGo conditions, half (50%) of the trials were congruent and the other half (50%) were incongruent. The order of trials in each block was randomized. Prior to the actual experiment, all participants completed a standardized practice run of 16 trials (without reward) to familiarize with the task.

Half of our participants performed the standard experimental paradigm (control), the other half performed a version to which we added the prospect of reward for good performance (reward group). Before the recording started, all participants were instructed to respond as fast and accurately as possible, so that they only rarely saw the speed-up sign appear. The reward group was additionally informed that each correct response execution (Go trials) or response omission (NoGo trials) could earn them a fixed monetary bonus that would be added up and paid as a reward for good performance on top of the standard monetary compensation in the end. They were also informed that they would only receive a bonus if the correct Go response was recorded before the speed-up sign (e.g., “Faster!”) appeared. The performance-based reward was calculated based on the following rules: participants could earn a reward for Go trials in which the correct response was recorded within 500 ms or less after stimulus presentation, and for NoGo trials in which no response was recorded post-stimulus presentation. Specifically, with each correct response or response omissions participants could earn 0.008 €, resulting in a maximal reward of 1 € per block and an overall reward sum of (max.) 6 € for the entire experiment.

After each block, all participants could take a self-timed break (i.e., to rest their eyes), and resume via button press. Moreover, for the participants of the reward group, the accumulated bonus amount of the last block, as well as the overall winning/reward sum (summation of all block earnings) was displayed on the screen during the break. The experiment took approximately 30 min to complete in both groups.

### EEG recording and analysis

During the experiment, a high-density EEG was recorded from 60 equidistant Ag–AgCl electrodes using the BrainVision Recorder software (Version 2.2) and a QuickAmp amplifier (Brain Products GmbH, Gilching, Germany). The reference electrode was positioned at Fpz and a ground electrode was placed at the coordinates θ = 58, φ = 78. All electrode impedances were kept below 10 kΩ. The EEG data was initially recorded at a sampling rate of 500 Hz and offline down-sampled to 256 Hz. EEG preprocessing was performed using Automagic^45^ and EEGLAB^46^ on Matlab 2019a (The MathWorks Corp.). First, flatlined channels (i.e., channels that showed activity below 5 µV for more than 5 s) were removed, the EEG data was re-referenced to the average reference, and missing channels were interpolated. Following this step, the PREP preprocessing pipeline^47^ and the EEGLAB clean_rawdata() pipeline were applied to remove irregular artifacts and detect noisy channels. In short, the PREP pipeline removes line noise (for data recorded in Europe: 50 Hz) by means of a multi-taper algorithm and after removing contaminations by bad channels, a robust common average reference is applied. The clean_rawdata() pipeline detrends the EEG data using a FIR high-pass filter of 0.5 Hz (order 1286, stop-band attenuation 80 dB, transition band 0.25–0.75 Hz). Furthermore, epochs with irregularly strong power (> 15 standard deviations relative to calibration data) were reconstructed using Artifact Subspace Reconstruction (ASR; burst criterion: 15^48^). Time windows that could not be reconstructed were removed and an additional bad channel detection was applied using high and minimum variance criterion. Furthermore, a low-pass filter of 40 Hz (sinc FIR filter; order: 86^49^) was applied. For EOG artifacts, a subtraction method was used (EOG Regression^50^). Moreover, the Multiple Artifact Rejection Algorithm (MARA^51,52^), that automatizes the process of independent component analysis (ICA), was applied to detect remaining artifacts in the data. Finally, missing and removed channels were interpolated using a spherical method. After visual inspection of the preprocessed EEG recordings, the EEG data were segmented into the corresponding four trial conditions (in/congruent Go/NoGo trials) using the BrainVision Analyzer software (Version 2.2, Brain Products GmbH). All of those segments were locked onto the stimulus onset. In Go conditions, only trials with correct responses within a response window of 100 to 1300 ms relative to the onset of the stimulus were considered valid trials. In NoGo conditions, valid trials were defined as trials in which no response was recorded within 0 to 1700 ms after stimulus onset. Each segment had a length of 2000 ms, starting 500 ms prior to stimulus onset until 1500 ms post-stimulus presentation. To remove any remaining artifacts, an automatic artifact rejection inspection was applied (rejection criteria: amplitude difference of more than 200 µV within 200 ms; amplitudes above 100 µV; activity below 0.5 µV in 100 ms). Finally, a baseline correction was applied to the time window of − 300 to 0 ms prior to the stimulus onset and the single-trial segmented EEG data was exported for further analysis.

### Residue iteration decomposition (RIDE)

In a next step, RIDE was run on the segmented and baseline-corrected single-trial EEG data by using the “RIDE toolbox” (available at http://cns.hkbu.edu.hk/RIDE.htm) and applying previously established protocols^31,33^. The RIDE temporal decomposition was conducted separately for each single electrode channel^35^. In short, RIDE decomposes the ERP signal into a stimulus-locked component cluster (S), a response time locked cluster (R) and a non-marker-locked intermediate cluster (C)^32,53^. RIDE presumes that in each single EEG trial, these three components (S, C and R) with variable intercomponent delays are linearly superimposed. Importantly, each component is associated with specific aspects of stimulus- and response-related processes. The S-cluster reflects stimulus-related attentional/perceptional processes, the R-cluster was shown to reflect response (execution)-associated processes and the central C-cluster (with variable latency) is representing stimulus evaluation and response selection processes^32,54^. However, there is no correct motor response for NoGo trials. Therefore, it is not possible to reliably estimate the R-Cluster in correct NoGo trials^53^. Against this background, only the S-cluster (reflecting stimulus-related processes^31^) and the central C-cluster were computed in the current study in order to disentangle stimulus-related and central aspects of processing and in order to account for the expected intra-individual variability within trials. After visual inspection of the pre-processed EEG data, time markers for the S-cluster were set from − 200 ms prior to 450 ms after stimulus onset. For the C-cluster, the estimation time window was set to 150–800 ms after stimulus presentation. Based on the initial time-markers, RIDE decomposes the ERP components in an iterative way by employing *L1*-norm minimization (i.e., obtaining median waveforms), so that the C-cluster latencies were initially estimated and iteratively improved. The latency-corrected ERP waveforms obtained from the RIDE algorithm enable us to clearly ascribe the underlying processes to specific cognitive processing steps and can be interpreted analogous to classical ERPs. For further mathematical and methodological details on the established RIDE procedures, please refer to previous publications by its creators^32,35^. Electrodes and time windows for the quantification and analysis of the resulting clusters were selected following visual inspection of the scalp topographies as well as electrode signals and are highly comparable with previous studies using the same experimental paradigm^26,42^. The electrodes and time windows in which amplitudes were quantified are summarized in Table 1.

**Table 1:** RIDE ERPs (for each cluster), corresponding electrodes and time windows (in milliseconds) in which the amplitudes were quantified as the mean activity in a given condition.

RIDE Cluster	RIDE ERP	Electrode(s)	Time window (group)	Condition
S	S-N1	P7, P8	150–190 ms (both groups)	Go & NoGo condition
	S-P2	P7, P8	270–315 ms (both groups)	Go condition
		P7, P8	260–305 ms (both groups)	NoGo condition
	S-N2	Cz	245–290 ms (reward group)	Go & NoGo condition
			255–300 ms (control group)	
		FCz	260–305 ms (reward group)	Go & NoGo condition
			280–325 ms (control group)	
C	C-P3	Cz	395–460 ms (both groups)	Go condition
			435–500 ms (both groups)	NoGo condition
		FCz	400–465 ms (both groups)	Go condition
			440–505 ms (both groups)	NoGo condition

### Ethics declaration

The study was approved by the Ethics Commission of the Medical Faculty of the TU Dresden (SR-EK-8012020) and conducted in accordance with the declaration of Helsinki.

## Source localization

Source localization analyses were conducted to examine differences between the experimental groups regarding the estimated functional neuroanatomical regions that are relevant to the modulation of the interplay of automaticity and cognitive control during response inhibition. For that, the sLORETA (standard low resolution electromagnetic tomography^55^, software package was used. sLORETA provides a linear solution to the inverse problem and estimates sources without localization bias^55,56^. Cortical and hippocampal gray matter is divided into 6239 voxels at 5 × 5 × 5 mm spatial resolution and used as the solution space. For each voxel, the standardized current source density (CSD) was estimated and log-transformed. We contrasted the decomposed EEG-data (C-cluster) Simon NoGo effect (i.e., the difference between congruent and incongruent NoGo trials) between reward and control group using the sLORETA statistical non-parametric mapping (SnPM) tool. Based on this, significant differences in source activity generators were determined performing 2000 permutations. The digitized structural MRI template used in the current study was the MNI152 head model template. Results from the sLORETA analysis located in the MNI template (corrected for multiple comparisons, *p* < .05) are shown in the results section.

## Statistical analysis

The behavioral data (i.e., accuracy and reaction times) and the neurophysiological data (RIDE cluster-derived ERP amplitudes and standard ERP amplitudes) were analyzed using IBM SPSS Statistics for Windows, version 28.0.1.1 (IBM Corp., Armonk, N.Y., USA). Separate repeated-measures ANOVAs with “group” (reward vs control group) as between-subject factor and “condition” (Go vs NoGo) and “congruency” (congruent vs incongruent) as within-subject factors were run. The ANOVAs for the neurophysiological data additionally included the factor “electrode” as within-subject factor. Prior to running the ANOVAs, outliers were identified using the SPSS built-in exploratory outlier analysis. Whenever a case was identified as an extreme outlier (i.e., either 3rd quartile + 3*interquartile range, or 1st quartile—3*interquartile range) in a given measure for two or more experimental conditions, that case was no longer considered representative of the sample and was entirely excluded from the ANOVA for that respective measure. In case outliers were detected in the behavioral data, the respective participants were also excluded from subsequent analyses of the neurophysiological data (but not vice versa. Yet, excluding the behavioral data of participants with statistical outliers in any neurophysiological measure would not have altered the obtained pattern of significant vs non-significant effects in a relevant manner). Whenever appropriate, Greenhouse–Geisser correction was applied to the reported ANOVA values. Significant main effects or interactions were examined with post-hoc ANOVAs and post-hoc t-tests. All variables were tested for normal distribution using Kolmogorov–Smirnov tests. When the assumption of normal distribution was violated, significant main effects and post-hoc tests run on these measures were additionally confirmed using non-parametric Wilcoxon signed-rank tests / Mann–Whitney U-tests.

Furthermore, Pearson’s correlation coefficients were computed to measure the relationship/association between the RIDE decomposed ERPs of the NoGo condition and the behavioral Simon NoGo effect. In case there was evidence for a significant correlation, additional linear regression analyses were performed to investigate a possible causal relationship between NoGo ERPs quantified in the RIDE clusters and the behavioral Simon NoGo effect.

When there was a trend towards a significant main effect or interaction, (i.e., *p* < .150), add-on Bayesian analyses were conducted to examine the relative evidence for the H_0_ compared to the H_1_. Using the template by Masson^57^, Bayes Factors (BF_01_) were computed and interpreted as can be seen in the supplementary material (Table 1). In case of positive evidence for the alternative hypothesis, further exploratory post-hoc analyses were conducted.

For all descriptive statistics, the mean value and the standard error of the mean (SEM; measurement of variability) are reported.

## Results

### Exclusion criteria

As for the accuracy measure, no participant performed below 60% accuracy in two or more task conditions, or was identified as an extreme outlier (for details refer to the “Methods” section). Regarding the reaction time data, n = 1 participant of the reward group was identified as an extreme outlier and thus removed from all subsequent analyses of the behavioral and neurophysiological data. Considering the neurophysiological data, n = 2 participants of the control group were identified as extreme outliers in the S cluster N1 measure and therefore excluded from the analysis of the RIDE-decomposed N1. For the P3 quantified in the C cluster, n = 1 participant from the reward group was identified as an extreme outlier and thus excluded from this particular analysis.

A sensitivity power analysis (G*Power software, Version 3.1.9.7^58^) was run to determine the statistical sensitivity of the remaining (min. 74) participants. It showed that with n = 74 participants (split into two groups, across 4 conditions), we can reliably detect effect sizes as low as 2.9% of the explained variance (*η*^2^_*p*_ = 0.029) for within-between interactions (with a power of 95% and an alpha error probability of 5%). Evidence of studies employing the task at hand showed interactive effects with effects sizes of approximately ~ *η*^2^_*p*_ = 0.17–0.27^27,59,60^, and interactive effects including the group factor showed effect sizes between ~ *η*^2^_*p*_ = 0.035 and ~ *η*^2^_*p*_ = 0.259^26,27,59^, so that the study is likely sufficiently powered.

### Behavioral data

#### Task effects

The ANOVA analysis for accuracy revealed a main effect of condition (*F*_(1,73)_ = 53.712; *p* < .001; *η*^2^_*p*_ = 0.424), with overall higher accuracy in Go (95.45% ± 0.43) than in NoGo trials (89.60% ± 0.83). This result was confirmed by an additional Wilcoxon signed-rank test (*Z* = − 5.545, *p* < .001). Furthermore, there was a main effect of congruency (*F*_(1,73)_ = 20.807; *p* < .001; *η*^2^_*p*_ = 0.222), indicating higher accuracy in incongruent (93.21% ± 0.50) than in congruent trials (91.84% ± 0.59). An add-on Wilcoxon signed-rank test confirmed the statistically significant difference (*Z* = − 4.214; *p* < .001). Notably, there was an interaction of congruency x condition (*F*_(1,73)_ = 35.370; *p* < .001; *η*^2^_*p*_ = 0.326), which is the typical task effect usually observed in the Simon NoGo paradigm. Post-hoc paired t-tests indicated significant congruency effects in both Go trials (*t*_(74)_ = 2.587; *p* = .012; congruent = 96.03% ± 0.47; incongruent = 94.90% ± 0.53) and NoGo trials (*t*_(74)_ = − 6.346; *p* < .001; congruent = 87.73% ± 1.21; incongruent = 91.59% ± 0.84). The different direction of the Simon effect (congruent minus incongruent) in Go trials (0.56% ± 0.22) versus NoGo trials (− 1.93 ± 0.30) was confirmed to be meaningful/significant (*t*_(74)_ = 5.642; *p* < .001). Notably, this finding is consistent with previous studies using this experimental paradigm^26,61^, and was additionally confirmed by a Wilcoxon signed rank test (*Z* = − 5.030; *p* < .001).

**Figure 2:**
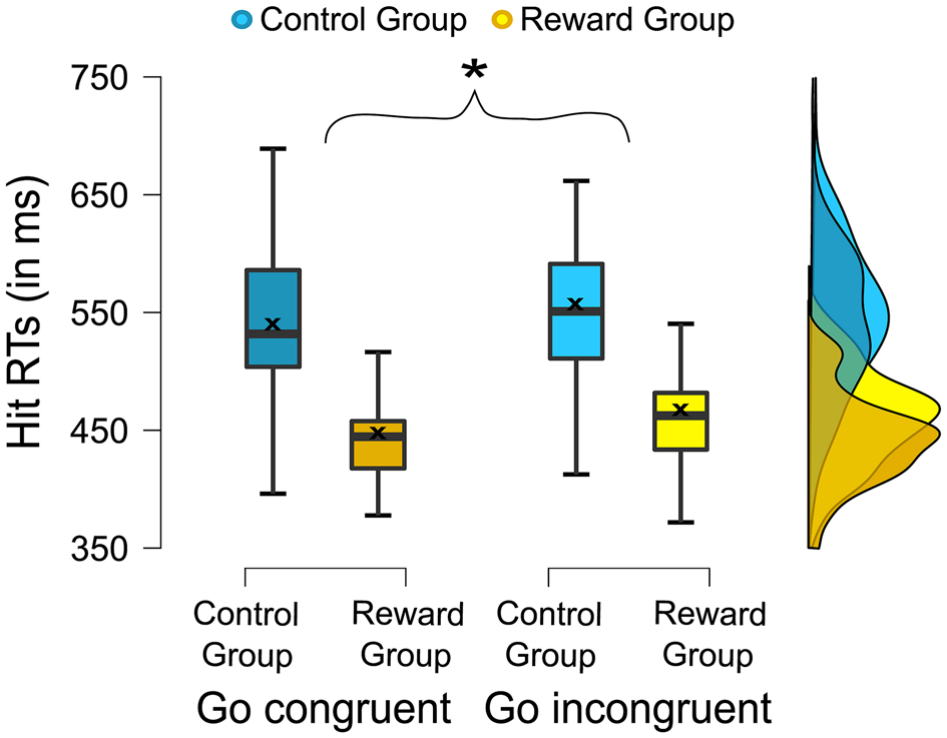
Behavioral results: Reaction times. The boxplots show the reaction times in milliseconds for correct (congruent and incongruent) Go trials. The “x” and the horizontal line inside the boxplots indicate the mean and median, respectively. The asterisk indicates significant differences, at *p* < .05, and the error bars represent the 95% confidence intervals. The cloud plot (right side) illustrates the data distribution.

For the Go reaction time (RT) measure (i.e., the time between target onset and correct Go response in ms), the obtained data is shown in Fig. 2*.* The ANOVA analysis revealed a main effect of congruency (*F*_(1,73)_ = 66.917; *p* < .001; *η*^2^_*p*_ = 0.478), showing that responses were faster in congruent (493.57 ± 6.64 ms) than in incongruent trials (510.81 ± 6.61 ms). This result was also confirmed by an add-on non-parametric test (*Z* = − 6.294; *p* < .001).

#### Group effects

The repeated measures ANOVA for accuracy further showed a main effect of group (*F*_(1,73)_ = 34.433; *p* < .001; *η*^2^_*p*_ = 0.321), indicating overall lower response accuracy in the reward group (89.44% ± 0.75) as compared to the control group (95.60% ± 0.74). An add-on Mann–Whitney U test confirmed the significant difference (U = 209.50; *Z* = − 5.230; *p* < .001). Moreover, there were significant interactions of condition x group (*F*_(1,73)_ = 19.600; *p* < .001; *η*^2^_*p*_ = 0.212) and of condition x congruency x group (*F*_(1,73)_ = 5.670; *p* = .020; *η*^2^_*p*_ = 0.072). Subsequent post-hoc tests separately investigating the magnitude of the Simon effect (congruent minus incongruent) in both conditions revealed a significantly larger/more negative Simon NoGo effect in the reward group (− 2.66% ± 2.97), as compared to the control group (− 1.22% ± 2.06) (*t*_(63.822)_ = 2.446; *p* = .017). This significance was confirmed by a Mann–Whitney U test (U = 458.50; *Z* = − 2.594; *p* = .009). In contrast to this, there was no significant group difference in the Simon effect of the Go condition (*t*_(47.522)_ =  − 1.212; *p* = .231). Other interactions with the group factor did not reach significance (*F*_(1,73)_ = 2.155; *p* = .146). For the Go RTs, there was a main effect of group (*F*_(1,73)_ = 58.695; *p* < .001; *η*^2^_*p*_ = 0.446), with faster responses in the reward group (452.10 ms ± 9.31) than in the control group (552.28 ms ± 9.18). The interaction of group x congruency did not reach significance for Go RTs (*F*_(1,73)_ = 0.828; *p* = 0.366).

**Figure 3:**
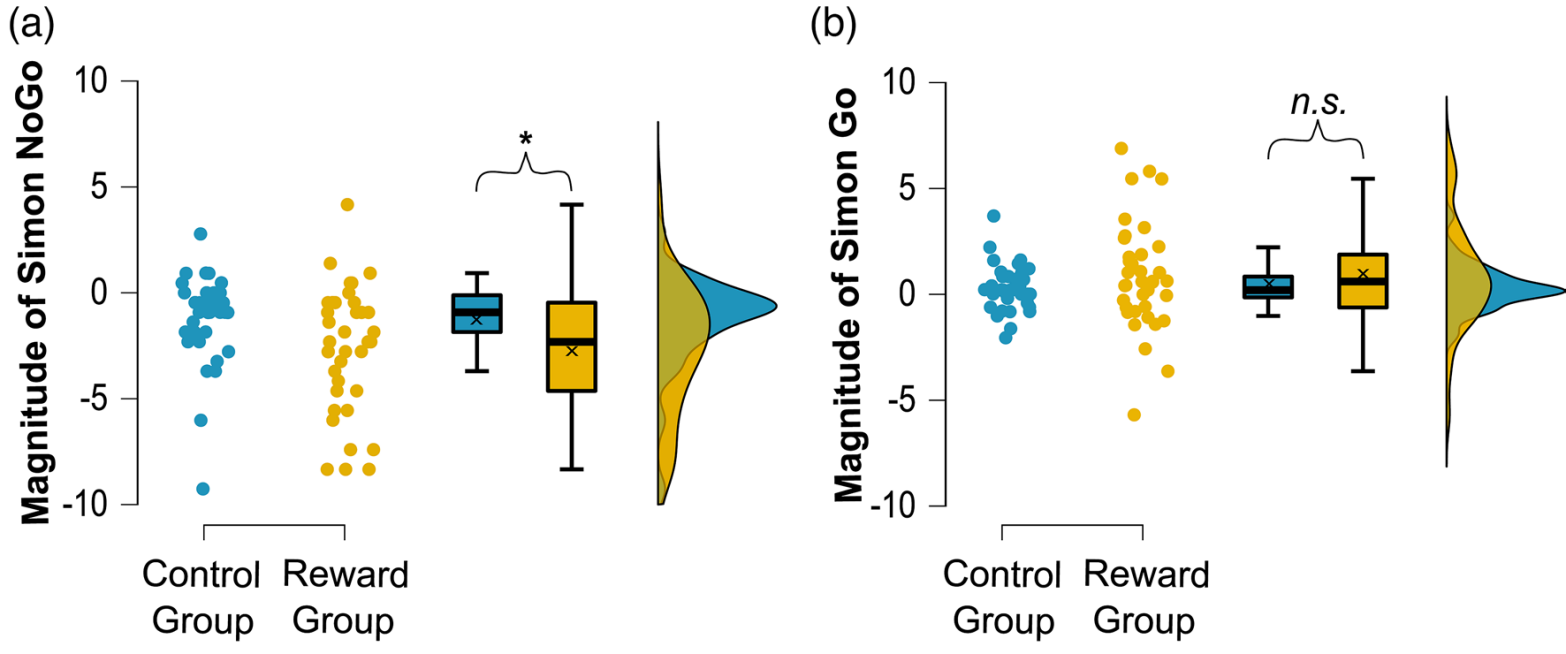
Behavioral results: Accuracy. The “x” and the horizontal line inside the boxplots indicate the mean and median, respectively. The asterisk indicates significant differences at *p* < .05, and the error bars represent the 95% confidence intervals. The raincloud plots illustrate the data distribution (**a** and **b** right side) and data points (**a** and **b** left side). (**a**) Magnitude of the Simon NoGo effect (correct congruent MINUS incongruent NoGo trials) based on the percentage of correct response omissions. (**b**) Simon Go Effect (correct congruent MINUS incongruent trials) based on the percentage of correct response executions.

For the accuracy measure (i.e. the Simon Go and the Simon NoGo effect), the obtained data for both groups is shown in Fig. 3.

#### Neurophysiological data: RIDE decomposition

In order to allow the reader to focus on the main research question, only main and interaction effects including the “group” factor are reported in the main manuscript. Details on all main and interaction effects that do not include the “group” factor can be found in the supplementary materials (for general information on how the different task conditions affect common event-related potentials, please also refer to previous publications on the Simon NoGo task^26,42^).

#### S-cluster

**Figure 4:**
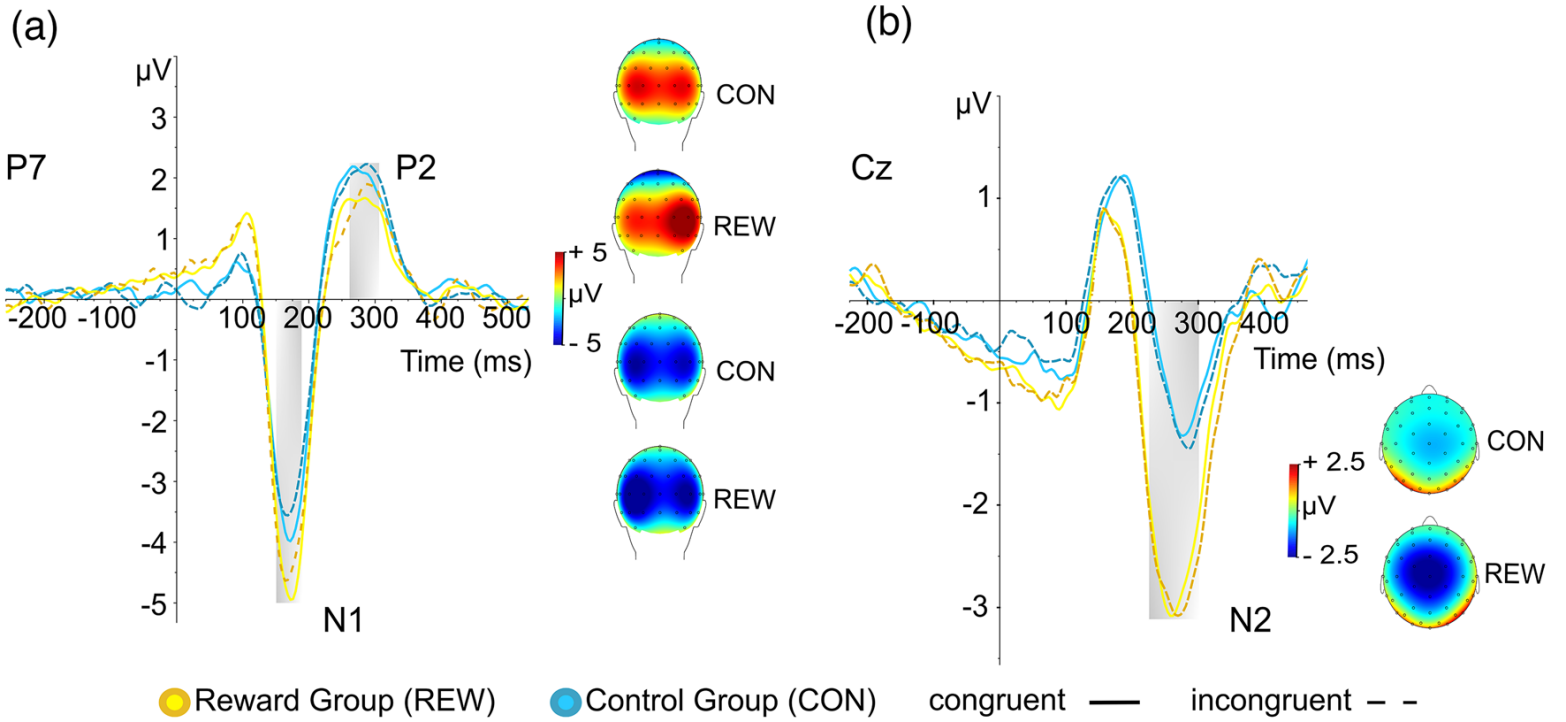
RIDE decomposed S-cluster NoGo ERPs and corresponding scalp topography maps. The color coding of the decomposed ERP signals indicates the different experimental groups, i.e., control group (blue) and reward group (yellow). The different lines indicate congruent NoGo trials (solid line) and incongruent NoGo trials (dashed line). Timepoint zero denotes the target letter onset. (**a**) The decomposed NoGo-N1 and NoGo-P2 for both groups are depicted at electrode P7 with corresponding scalp topography maps on the right (top P2, bottom N1). (**b**) The decomposed NoGo-N2 at electrode Cz is shown with corresponding scalp topography maps on the bottom right. The scalp topography maps show the voltage distribution in the time windows (area marked with grey boxes) in which the amplitudes were quantified as the average electrode activity (see Table 1 for details).

The analyzed S-cluster ERPs are illustrated in Fig. 4.

*N1.* The repeated measures ANOVA for the S-cluster N1 revealed a main effect of group (*F*_(1,71)_ = 6.820; *p* = .011; *η*^2^_*p*_ = 0.088), showing significantly larger N1 amplitudes in the reward group (− 4.352 μV ± 0.346), as compared to the control group (− 3.066 μV ± 0.351). The interaction of congruency x group (*F*_(1,71)_ = 3.261; *p* = .075; *η*^2^_*p*_ = 0.044) did not reach significance, and an add-on Bayesian analysis provided anecdotal evidence for the H_0_ (*BF*_01_ = 1.66). No other interactions with the factor group were evident (all *F* < 1.522; all *p* > .221).

*P2.* The analysis for the S-cluster P2 showed an interaction of group x electrode (*F*_(1,73)_ = 8.351; *p* = .005; *η*^2^_*p*_ = 0.103). In the reward group, post-hoc paired t-tests revealed significantly larger (*t*_(36)_ =  − 3.736, *p* < .001) P2 amplitudes at electrode P8 (2.659 μV ± 0.277) than at electrode P7 (1.439 μV ± 0.299). This lateralization effect was not found in the control group (*p* = .938). Other main or interaction effects with the factor group did not reach significance (all *F* < 1.662; all *p* > .201).

*N2.* For the S-cluster N2, the repeated measures ANOVA showed a main effect of group (*F*_(1,73)_ = 12.662; *p* < 0.001; *η*^2^_*p*_ = 0.148), revealing larger N2 amplitudes in the reward group (− 2.634 μV ± 0.279), as compared to the control group (− 1.237 μV ± 0.276). As the assumption of normal distribution for the N2 component was violated, an additional Mann–Whitney U test was performed, which was significant (U = 401.0; *Z* = − 3.20; *p* = 0.001). Furthermore, there was a significant interaction of electrode x group (*F*_(1,73)_ = 5.139; *p* = 0.026; *η*^2^_*p*_ = 0.066). Post-hoc t-tests indicated a significantly larger difference (Cz minus FCz) in N2 amplitudes between Cz and FCz in the reward group (− 0.621 μV ± 0.167), as compared to the control group (− 0.022 μV ± 0.204) (*t*_(73)_ = 2.267, *p* = .026). An additional Mann–Whitney U test confirmed the results (U = 498.00; *Z* = − 2.172; *p* = .030). Further interactions with the factor group did not reach significance (all *F* < 1.315; all *p* > .255).

#### C-cluster

**Figure 5:**
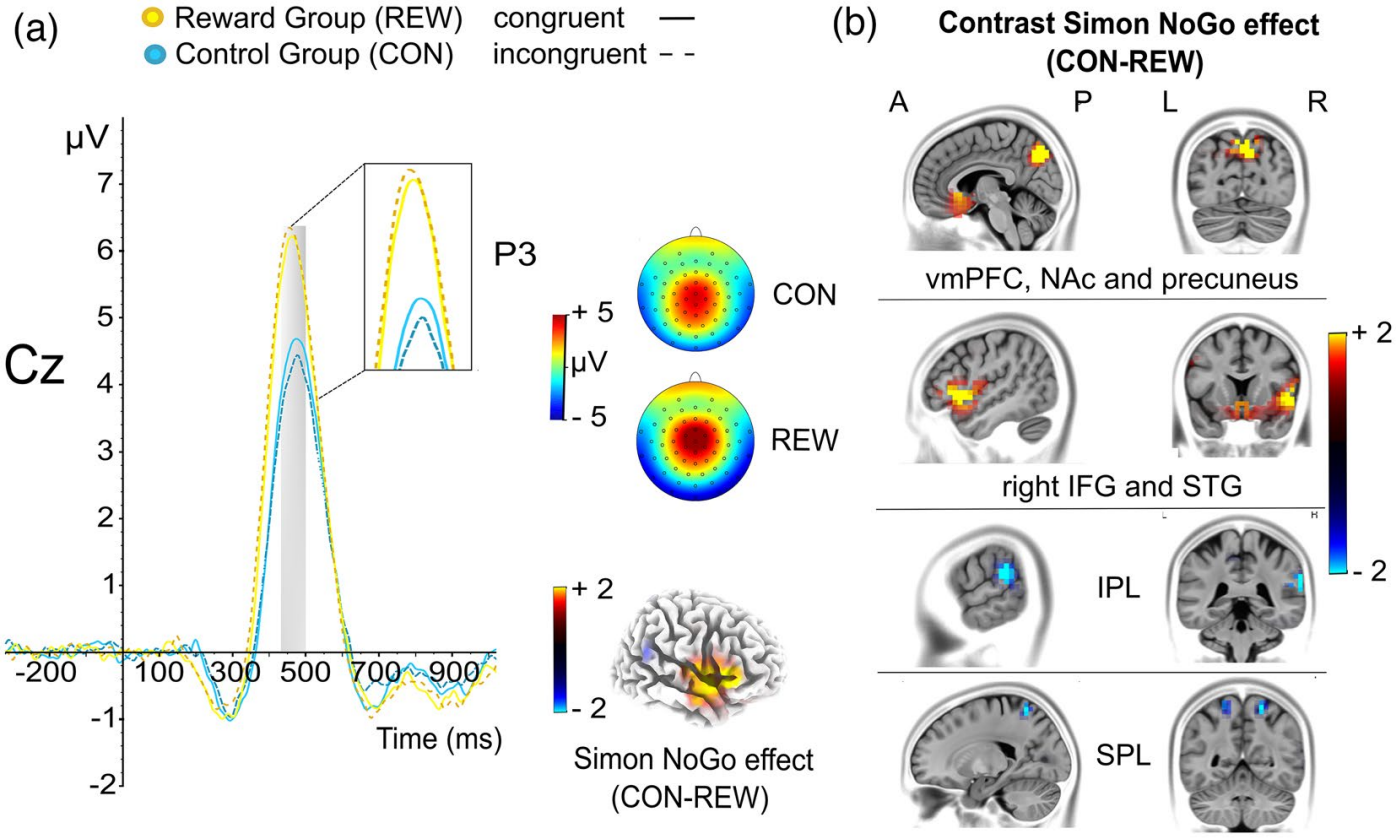
RIDE decomposed C-cluster NoGo-P3 and sLORETA maps. (**a**) The distinct colors of the decomposed ERP signals indicate the different experimental groups, i.e., control group (blue) and reward group (yellow). The different lines indicate congruent NoGo trials (solid line) and incongruent NoGo trials (dashed line). The scalp topography maps show the voltage distribution in the P3 time window (area marked with a grey box) in which the amplitude was quantified as the average electrode activity (see Table 1 for details). Additionally, a 3D volume shows the sources of maximal differences (contrast of the Simon NoGo effect) on the right hemisphere between control and reward group. (**b**) sLORETA-derived maps show the contrast of the Simon NoGo effect (congruent MINUS incongruent NoGo trials), indicating the sources of maximal activation differences between the control group and reward group in the NoGo-P3 time window. *vmPFC* ventromedial prefrontal cortex, *NAc* nucleus accumbens, *IFG* inferior frontal gyrus, *STG* superior temporal gyrus, *IPL* inferior parietal lobule, *SPL* superior parietal lobule.

The analyzed C-cluster ERP is illustrated in Fig. 5.

*P3.* For the frontal C-cluster P3, the repeated measures ANOVA revealed a main effect of group (*F*_(1,72)_ = 10.969; *p* = .001; *η*^2^_*p*_ = 0.132), indicating overall larger C-P3 amplitudes in the reward group (3.935 μV ± 0.354), as compared to the control group (2.298 μV ± 0.345). There was also a significant interaction of condition × electrode × group (*F*_(1,72)_ = 5.104; *p* = .027; *η*^2^_*p*_ = 0.066). Post-hoc tests revealed that for the control group, the interaction of condition × electrode was not significant (*F*_(1,37)_ = 0.942; *p* = .338; *η*^2^_*p*_ = 0.025). For the reward group however, the analysis revealed a significant interaction of condition × electrode (*F*_(1,35)_ = 15.123; *p* =  < .001; *η*^2^_*p*_ = 0.302). Further post-hoc tests showed that for the reward group, there were significantly larger condition differences (NoGo minus Go) at electrode FCz (3.735 μV ± 0.372) as compared to Cz (2.786 μV ± 0.368) (*t*_(35)_ = − 3.889; *p* < .001).

The interaction of condition × congruency × group (*F*_(1,72)_ = 2.747; *p* = .102; *η*^2^_*p*_ = 0.037) did not reach significance, but add-on Bayesian analysis indicated moderate evidence for the H_1_ (BF_01_ = 0.23). Subsequent exploratory post-hoc analyses revealed no interaction effects for Go trials (F = 0.223; *p* ≥ .638, but an interaction of congruency × group (*F*_(1,72)_ = 4.672; *p* = .034; *η*^2^_*p*_ = 0.061) in NoGo trials. In line with this, post-hoc t-tests revealed a significantly more negative P3 Simon NoGo effect (*t*_(72)_ = 2.162; *p* = .034) in the reward group (− 0.108 μV ± 0.162) as compared to the control group (0.367 μV ± 0.149). An add-on source localization analysis (sLORETA) showed that modulations (contrasting the Simon NoGo effect of the control and reward group) in the P3 quantification time window were associated with activation differences in the following regions: right inferior parietal lobule (IPL, BA40), superior parietal lobule (BA7), right inferior frontal gyrus (IFG; BA 47), right superior temporal gyrus (STG; BA 38), ventromedial prefrontal cortex (vmPFC), nucleus accumbens and the precuneus (BA7). No other interaction with the factor group was evident in the ANOVA for the frontal C-cluster P3 (all *F* < 1.524; all *p* > .221).

#### Decomposed EEG data as a predictor of the behavioral Simon NoGo Effect

Taken together, the most important behavioral and neurophysiological group differences (i.e., those that were not merely main group effects) were restricted to the NoGo condition. Consequently, we decided to limit all subsequent analyses to the NoGo condition. We set out to determine whether there is a linear relationship between the behavioral NoGo performance (mean NoGo accuracy and mean Simon NoGo effect) and neurophysiological NoGo effects/ERPs quantified in the RIDE clusters. Overall, the conducted correlation analyses (see Table 2 for details) indicated clear differences between the control and the intervention/reward group: In the control group, there was a significant positive correlation between the behavioral Simon NoGo effect and the mean S-cluster NoGo N1 at electrode P7, as well as the mean S-cluster NoGo P2 at electrode P7. Furthermore, the control group showed a significant positive correlation between the mean accuracy in NoGo trials and the S-cluster N1 Simon NoGo effect at electrode P7. In the reward group, a significant positive correlation between the mean accuracy in NoGo trials and the S-cluster P2 Simon NoGo effect (at electrode P7) was shown. For the control group, only a trend towards a negative correlation between the mean accuracy in NoGo trials and the S-cluster P2 Simon NoGo effect at electrode P7 was observed. This was however not confirmed with Bayesian analysis, which indicated substantial evidence for the H_0_ (BF_01_ = 4.854)_._ Additionally, there seemed to be a trend towards a positive correlation between the behavioral Simon NoGo effect and the C-cluster P3 Simon NoGo effect (at electrode Cz) for the reward group, but not for the control group. An add-on Bayesian correlational analysis for the reward group however indicated anecdotal evidence for the H_0_ (BF_01_ = 1.453).

**Table 2:** Pearson's correlation of RIDE decomposed ERPs and accuracy measures of the NoGo condition.

RIDE cluster/behavior	Control group		Reward group		Control group		Reward group	
	NoGo mean accuracy				Simon NoGo effect			
	*r*	*p*	*r*	*p*	*r*	*p*	*r*	*p*
N1 P7 mean NoGo	0.248	.144	− 0.063	.711	0.373*	.025	− 0.127	.453
N1 P7 Simon NoGo effect	−0.333*	.047	0.144	.394	− 0.143	.405	0.059	.727
P2 P7 mean NoGo	0.134	.423	0.164	.331	0.322*	.049	− 0.191	.258
P2 P7 Simon NoGo effect	− *0.312*	.056	0.334*	.044	− 0.165	.321	0.154	.364
P3 Cz mean NoGo	− 0.110	.511	0.196	.253	0.123	.463	0.002	.990
P3 Cz Simon NoGo effect	0.026	.877	0.145	.400	− 0.037	.826	*0.309*	.067

*Statistically significant at *p* < .05 level.

Significant values are in [bold and italics].

Nevertheless, an established linear association does not allow to postulate causality. To further investigate the assumed causal relationship, regression models were computed for all RIDE-Cluster ERPs that significantly correlated with the behavioral NoGo performance (i.e., mean accuracy in NoGo trials and Simon NoGo effect; see Table 2). Specifically, we set out to evaluate whether the amplitude of the NoGo trial ERPs quantified in the RIDE cluster are significant predictors of the behavioral performance in NoGo trials. All regression coefficients are summarized in Table 3. Using mean accuracy in NoGo trials as the dependent variable, the regression models yielded the following results: For the control group, variations in mean accuracy in NoGo trials were shown to be significantly predicted by the magnitude of the S-cluster N1 Simon NoGo effect (at electrode P7; *F*_(1,34)_ = 4.252; *p* = .047). This indicates that for the control group, lower mean NoGo accuracy is evident as the N1 Simon NoGo effect (congruent minus incongruent) becomes more positive. For the reward group, the mean accuracy in NoGo trials was shown to be significantly predicted by the S-cluster P2 Simon NoGo effect (at electrode P7; *F*_(1,35)_ = 4.383; *p* = .044). This means that the regression equation predicts that a smaller (i.e., less negative) S-cluster P2 Simon NoGo effect is associated with higher mean accuracy in NoGo trials. Additionally, the linear regression analysis showed that both the mean S-cluster NoGo N1 at electrode P7 (*F*_(1,34)_ = 5.499; *p* = .025) and the mean S-cluster NoGo P2 amplitude at electrode P7 (*F*_(1,34)_ = 4.154; *p* = .049) predicted the magnitude of the behavioral Simon NoGo effect in the control group. Accordingly, a smaller/less negative Simon NoGo effect is evident as N1 amplitudes decrease (i.e., become less negative) and P2 amplitudes increase in the control group. For the reward group, none of the ERPs quantified in the RIDE-clusters could explain the magnitude and direction of the observed behavioral Simon NoGo effect. Corresponding graphs illustrating the data presented in Table 3 are provided in the supplementary material.

**Table 3:** Regression coefficients for predicting the behavioral performance in the NoGo condition.

Dependent Variable	Predictor	Control group			Reward group		
		B	R^2^	F	B	R^2^	F
Mean NoGo accuracy	N1 P7 Simon NoGo effect	− 2.090	0.111	4.252	1.755	0.021	0.744
	Constant	93.819			85.040		
Mean NoGo accuracy	P2 P7 Simon NoGo effect	− 2.242	0.098	3.895	2.970	0.111	4.383
	Constant	94.226			85.102		
Simon NoGo effect	N1 P7 mean NoGo	0.448	0.139	5.499	− 0.146	0.016	0.575
	Constant	0.097			− 3.301		
Simon NoGo effect	P2 P7 mean NoGo	0.288	0.103	4.154	− 0.301	0.036	1.323
	Constant	− 1.817			− 2.163		

## Discussion

The aim of the current study was to investigate whether the prospect of a reward for good performance modulates the interplay of automaticity and cognitive control during response inhibition. Therefore, the neurophysiological processes that reflect the interaction of reward prospect and cognitive control were assessed using a rewarded Simon NoGo Task. We further set out to determine whether relevant neurophysiological processes could predict successful motor response inhibition in the context of controlled and automatic response selection processes. Most importantly, the current study shows that reward prospect increases the influence of the interactive effects of automatic and controlled processes on response inhibition (i.e., reflected by a larger Simon NoGo effect), but not on response selection. Notably, stimulus–response translation processes (as reflected by the NoGo-P3) paralleled the reward-specific modulation of the behavioral interference effect. Add-on source localization analyses revealed underlying activation differences in the inferior frontal and somatosensory cortex. Additionally, our results indicate a correlative relationship between early attentional processes (as reflected by the N1) / resource allocation processes (as reflected by the P2) and successful motor response inhibition.

Well in line with previous findings, we found inverse effects of S-R congruency. That is, congruent S–R relations which allow to rely on automatic response selection processes (i.e., “direct-route”) were beneficial for response selection, but detrimental when response inhibition was required. In contrast, incongruent S-R relations increase the need for cognitive control to overcome the pre-potent automatic response tendencies, which is beneficial for response inhibition^26^. In incongruent NoGo trials, the interaction between incorrect automatic response tendencies and the “indirect route” (i.e., controlling the influence of automatic response tendencies) facilitates inhibitory control, leading to the inverted Simon effect in NoGo trials. Our results show that reward prospect had particularly detrimental effects on inhibitory performance in the more automatized (congruent) NoGo trials. It is therefore possible that reward prospect increased the automaticity of pre-potent responses and thereby raised the need for inhibitory control, and the associated likelihood of commission errors.

Moreover, the current data indicates an overall performance-impeding effect of reward prospect that was reflected in lower performance (i.e., response accuracies) in the reward group, as compared to the control group. Our results further suggest that the groups differed in the employed speed-accuracy trade-off strategy (SATS), which is an omnipresent process regulating the competing demands in decisional processes^62^. Based on this, higher false alarm rates in the reward group are likely the result of a liberal/less informed decision strategy leading to faster and less accurate responses^63^. Contrastingly, the control group showed a more cautious/informed strategy, that is reflected in slower but more accurate responses. The results for both groups indicate the characteristical disadvantages for response inhibition when processing is mediated via the automatic route^26^, i.e., more false alarms in congruent as compared to incongruent NoGo trials. In the reward group, the typical task effect was amplified by SATS to a larger extent (due to the monetary reward prospect), than in the control group. Importantly, there is no response time (as participants are required to withhold their responses), based on which a “speed” and potential accuracy-tradeoff could have been determined in NoGo trials. In principle, it would be possible to use the reaction times of false alarms committed in this condition, however these were (and typically are) too scarce, varied and non-informative to base reliable analyses/assumptions on them. The possibility exists that the applied reward-rule/instructions (i.e., rewards only for accurate response selection/omissions before the speed-up sign appeared), contributed to an emphasis on speed over accuracy, which in turn reduced the quality of stimulus(-response) processing^64,65^. Additionally, the ratio of Go versus NoGo trials (i.e., 7:3) may have further increased the emphasis on response execution and increased the willingness of the reward group to trade response speed in Go trials at the cost of correct response selection/omission in NoGo trials. As suggested by previous evidence^37,66^, this tilted distribution of trials was essential to induce strong automated response tendencies, consequently imposing high demand on inhibitory control processes. Thus, different intertrial intervals and/or Go versus NoGo ratio configurations may not lead to the same results^37,66^. In accordance with the current results, liberal SATS have been shown to decrease response times and lead to lower response accuracies in Simon Tasks^63^. Furthermore, and in agreement with previous research (using flanker and decision-making tasks^67,68^), participants who were rewarded for good performance responded significantly faster than participants who had no reward prospect. Altogether, it hence seems that the performance-based monetary reward in combination with implicit (i.e., verbal instructions) and explicit (i.e., speed-up sign) response deadlines fostered an exploitation of time at the cost of response accuracies^69^.

Earlier studies have shown that Simon effects are stronger for fast responses than for slow responses (for review see^59^). In the current study, however, differences between the groups in the magnitude of the accuracy-based Simon effect were only found when response inhibition was required. According to the activation-suppression model^41^, faster response selection tendencies in the reward group (i.e., before the response conflicts had been fully processed), may have led to the overall observed higher rate of false alarms. However, this does not fully explain the larger interference effect in NoGo trials. On another account, action inhibition success has been described as a race between two competing processes, in which the timing of go and stop processes is the crucial determinant of successful inhibition^71^. Consequently, the stopping process needs to be completed in time to interfere with the ongoing go process and therefore for inhibition to be successful. Based on the horse-race model^71,72^, the current results indicate that go processes were too fast for inhibitory processes to be completed in time. In the employed experimental paradigm, the NoGo condition required both “interference inhibition” and “action inhibition”. The latter was especially the case in congruent NoGo trials in which the level of automaticity of pre-potent responses is high, thus increasing the inhibitory requirements. Taken together, it is therefore reasonable to conclude that the overall emphasis of the reward group on fast (less accurate) response execution did not only increase the conflict effects, but also left no time for action inhibition to be completed in time to withhold erroneous responses. This was particularly detrimental for the more automatized congruent trials, leading to the observed larger Simon NoGo effect in the reward context.

On the neurophysiological level, we were able to shed light on the mechanisms and neuroanatomical structures underlying the impeding influence of reward prospect on interference effects during motor response inhibition. When there was a prospect of reward for good performance, successful inhibition of automatic response tendencies appears to be related to the strength and direction of the cognitive resource allocation conflict that arises between controlled versus automatic response contexts. The impeding effects of reward prospect on the ability to inhibit automatic pre-potent responses (i.e., Simon effect) were mirrored by the C-cluster, which reflects the intermediate processes between stimulus (S) encoding and response (R) selection^31,54^. The C-cluster is likely to be modulated by inhibition of pre-potent automated responses and to reflect conflict and increased cognitive load, which is mostly reflected in the (NoGo)-P3^31^. Our data provides substantial evidence that reward prospect increased the recruitment of cognitive resources needed for the resolution of response conflicts during response inhibition (reflected by a larger/more negative P3 Simon NoGo effect as compared to the control group), which is in accordance with a recent study^73^. However, it has also been suggested that the C-cluster may reflect a purposeful deceleration of motor processes^74^. Thus, the lower amplitude in the congruent NoGo trials may correspond to deficient “braking” of pre-potent motor responses in the condition where this would be most necessary^27^. Add-on source localization analyses in the P3 time window revealed that the reward group was characterized by higher activation differences in the right inferior parietal lobule (IPL; BA 40) and the superior parietal regions (SPL; BA 7). In the context of Go/NoGo tasks, IPL and SPL activation were suggested to reflect the difficulty to inhibit pre-potent responses^75^. Furthermore, the activity in both regions has been shown to be sensitive to response conflict modulations, with greatest activity for high conflict trials^76,77^. In accordance with the current results, the IPL has been shown to reflect SR-mappings and task-reward associations^78^. Taken together, we propose that the increased activation differences in the reward group in the IPL (BA 40) and SPL (BA 7) reflect the difficulty to inhibit the reward-triggered pre-potent automatic responses in the presence of a response conflict. Prefrontal circuits, especially those including the IFG, can interfere with subcortical action selection processes (via the subthalamic nucleus) through which automatic and prepotent response tendencies can be suppressed^24^. In line with this, the reward group had lower activity differences in the right IFG (BA 47) during the P3 time window (as compared to the control group). The rIFG is known to mediate inhibitory control processes^79^, thus the reward prospect may have triggered lack of ‘behavioral break’, and/or the cognitive resources invested in response inhibition were not sufficiently adapted to the degree of automaticity versus control in the selection of the pre-potent responses. Moreover, the reward group also displayed lower activity differences in the right STG (BA 22). Both the IFG and STG are core regions of the ventral attention network^80^, which is thought to facilitate the quick adjustments to sudden changes and has been suggested to facilitate response inhibition (i.e., by drawing attention to appropriate S-R mapping processes). The diminished activity differences in the right STG in the reward group may further indicate deficiencies in visual information processing as compared to the control group^81,82^. Higher activation differences in the control group were also shown in a cluster spanning the ventromedial prefrontal cortex (vmPFC) and the nucleus accumbens. Earlier studies suggested that activity implications in the nucleus accumbens and the strongly connected vmPFC reflect failures to engage in behavioral control (e.g., impulsivity or speeded responses)^23^. Importantly, the nucleus accumbens is known to be involved in action monitoring as well as the optimization of goal-directed behavior^83^, therefore diminished activity differences in the reward group may relate to a lack of response strategy adjustment, consequently leading to the observed behavioral effect. Together, we argue that the difference in the extent to which the interactive effects of controlled and automatic processes influenced response inhibition are largely due to processes related to the association of stimuli with appropriate responses (i.e., S-R mapping) found in the C-cluster. We further suggest that the overall larger C-cluster P3 in the reward context indicates increased cognitive processing needed for stimulus classification and conflict resolution during response inhibition through top-down inhibitory processes^84,85^. Additionally, the fronto-central P3 has commonly been associated with the evaluation of motor inhibition processes^86,87^, and decreases in amplitude have been related to impaired inhibitory performance through a lack of behavioral performance evaluation. The larger P3 amplitudes in incongruent NoGo trials possibly reflect a higher extent of control mediated via the “indirect route”, which supported inhibitory control and sustained behavioral performance evaluation. Consequently, incongruent NoGo trials were protected to a larger extent from the influence of reward-triggered speeded responses than the more “automatized” congruent NoGo trials. Yet, strong evidence for a causal relationship between the behavioral Simon NoGo effect and the C-cluster could not be established. This lack of correlation effects indicates that the central aspects of S-R mapping alone do not provide a sufficient explanation for the impeded response conflict resolution triggered by the reward prospect. It is therefore possible that the reflection of the behavioral effects in the NoGo trials by the fronto-central P3 indicates stronger behavioral performance evaluation in the reward context.

Interestingly, our results indicated that early attentional (i.e., S-cluster N1) and resource allocation (i.e., S-cluster P2^42^) related processes significantly modulate the interaction of automatic and controlled processes during response inhibition (with and without reward prospect). Notably, lower attention/sensory processing (smaller N1 amplitudes) of the target stimuli were associated with lower interference effects during response inhibition in the control group. Therefore, increased early sensory processing in the reward context may have led to a more active representation of the stimuli, increasing the difficulty to suppress pre-potent automatic responses. In line with this, it may be speculated that the reward-triggered dopamine release may have further increased this perceptual sensitivity^4^, which is in line with accounts suggesting that rewards influence attentional processes^18^. Yet again, a causal relationship could however not be established. Here we show that increases in attentional resource allocation (i.e., reflected by the S-cluster NoGo-P2 amplitudes) facilitated response inhibition performance in the absence of a reward for good performance, possibly due to top-down attentional processes preventing the formation of automatic response tendencies (leading to lower interference effects during response inhibition)^88^. In line with this argumentation, we found smaller activity differences in the precuneus (BA 7) in the reward group, likely reflecting lower top-down allocation of cognitive resources^88,89^. This further supports the notion that the larger activity difference in the control group, as compared to the reward group, may indicate a higher degree of top-down suppression of precuneal cue-reactivity^90^, leading to the observed lower interference effect during response inhibition^59^. In the reward context, successful response inhibition of automatic response tendencies appeared to be related to the strength and direction of the cognitive resource allocation conflict that arises between controlled versus automatic response contexts (reflected by the S-cluster P2-Simon NoGo effect). Previous evidence suggests that the NoGo-P2 reflects perceived difficulty to inhibit an automatic response, probably due to underlying conflicts/impairments in inhibitory control processes^91^. Thus, it seems that the previously reported P2-modulation^42^ is to some degree also present in the context of reward prospect for good performance, further underlining the importance of early resource allocation in contexts in which response inhibition in more automatic versus controlled contexts is required. In agreement with previous studies, increased resource allocation processes were recruited during response inhibition, where cognitive control demands are higher as compared to response selection trials^92^.

The current results further suggest that the perceived pre-response conflict (which is typical for this task^26^) and mental effort needed to generate correct responses increased when there was a reward prospect, as reflected in larger N2 amplitudes. Previously, a positive relationship between larger N2 amplitudes and improved response inhibition was suggested^93.^ In our study, however, rewarded individuals with larger N2 amplitudes made more errors and false alarms as compared to individuals from our control group. The results can be explained in the context of recent studies showing that the N2 is not specific to (motor) response inhibition, but arises whenever effort is increased (i.e., modulated by stimulus probabilities), or when a response conflict is detected^37^, and there is a need to overcome pre-potent response tendencies, that would otherwise yield incorrect responses^36^. Other evidence from stop signal tasks suggests that the N2 amplitude reflects successful (when compared to failed) stopping^94,95^. Notably, no linear relationship between behavioral performance and the differences in perceived response conflict prior to response selection (i.e., S-cluster N2) could be established.

It is possible that the current behavioral effects of reward prospect may have been in part due to the general emphasis of the reward group on speed over accuracy, leading to the observed improvements in response speed, but not in conflict resolution processes. Following this line or argument, larger interference effects would be expected in both Go and NoGo conditions. However, reward prospect only increased the magnitude of interference effects when response inhibition was required. We therefore argue that both the speed-accuracy trade-off and the reward-induced enhancement of attention to the implementation of goal-directed action execution affected performance. This may sound counterintuitive, yet evidence suggests that reward facilitates response preparation^96^, even before the response-indicating stimulus has been presented^97,98^. In the context of the task at hand, high automaticity of pre-potent motor responses is detrimental, especially when response inhibition is required in an automatic versus controlled response context. On another account, the observed behavioral results may have been due to opportunity cost of time shifts in the speed-accuracy tradeoff on trials that required more cognitive control (i.e., the more difficult response inhibition trials), in favor of speeded but less accurate responses^99^. Adding to this, it has been suggested that monetary reward can counteract behavioral conflict adaptation^16^, so that a lack of conflict adaptation may have contributed to the larger interference effect during response inhibition in the reward context. On the one hand, the apparent speed-accuracy trade-off might have been an intentional decision, possibly even a reward-maximizing strategy that participants employed as they noticed that in a larger proportion of trials responses had to be executed rather than inhibited. This may have been further increased by the inherent and easier S-R mapping between reward and response execution (as compared to inhibition)^100^. On the other hand, there were no monetary losses for incorrect response selection/omissions in the current task, which would have aided the signaling of need for adjustment of cognitive control processes. These factors may have equally contributed to the lack of behavioral, and conflict adaptation that rewarded participants showed in our study.

## Conclusion

Taken together, rewarded participants responded faster, but less cautiously, leading to overall lower behavioral response accuracies. Importantly, our findings show that a prospect of reward for good performance further increased the influence of interactive effects of automatic versus controlled processes during response inhibition (i.e., larger Simon NoGo effect as compared to the control group), but not during response selection (i.e., no differences in the regular Simon effect in Go trials). The reward-specific behavioral effect was reflected in distinct modulations of the neurophysiological data, also indicating a larger P3 Simon NoGo effect of the reward group. In the absence of a performance-contingent reward, but not in the reward context, attentional/resource allocation processes were found to predict the magnitude of the behavioral Simon NoGo effect. The current results thus underline SR-mappings as a crucial (but not sole) determinant of distinctive Simon effects during response inhibition. Importantly, shining light on possible underlying mechanisms of the reward-specific behavioral effect, the reward group showed different modulatory effects (associated with the larger P3 Simon NoGo effect) in the inferior frontal, parietal and somatosensory cortex. Specifically, rewarded participants showed lower activation differences in the rIFG, rSTG, vmPFC, NAc and precuneus. In contrast to this, the sources of the larger activation differences, compared to the control group, were the IPL and SPL. Considering the overall impeding effect of reward on behavioral performance, the current results add to the account of possible dysfunctional effects of reward incentives on cognitive and consequently on motor inhibitory performance. We further provide novel insights into the reward-triggered performance trade-offs and associated neuroanatomical structures.

## Supplementary Information

Supplementary Information.

## Data Availability

All study data are available upon request to Anna Helin Koyun (AnnaHelin.Koyun@ukdd.de).
