# Supplementary material for: Neurophysiological mechanisms underlying the differential effect of reward prospect on response selection and inhibition
Source: Sci Rep. 2023 Jul 5;13:10903. doi: 10.1038/s41598-023-37524-z (PMC10322977; doi:10.1038/s41598-023-37524-z)

Supplementary Materials

Neurophysiological mechanisms underlying the differential effect of reward prospect on response selection and inhibition

Anna Helin Koyun, Ann-Kathrin Stock, Christian Beste

Bayes Factor interpretation

Table S1. Bayes Factors (BF) Interpretation.

Bayes factor, BF_{01}	*Interpretation*
10 - 30	Strong evidence for the H_0 compared to the H_1.
3 - 10	Substantial evidence for the H_0 compared to the H_1.
1 - 3	Anecdotal evidence for the H_0 compared to the H_1.
1	No evidence.
1/3 - 1	Anecdotal evidence for the H_1 compared to the H_0.
1/10 – 1/3	Substantial evidence for the H_1 compared to the H_0.
1/30 – 1/10	Strong evidence for the H_1 compared to the H_0.

(Wagenmakers et al., 2011)

RIDE results

S-cluster

N1. For the N1 amplitude, the ANOVA revealed a significant main effect of condition ($F_{(1,71)} = 5.431$, $p = .023$, $\eta^2_p = 0.071$), showing more negative amplitudes in Go trials (-3.776 µV ± 0.242) than in NoGo trials (-3.641 µV ± 0.254). There were significant interactions of congruency x electrode ($F_{(1,71)} = 4.601$, $p = .035$, $\eta^2_p = 0.061$) and of condition x congruency x electrode ($F_{(1,71)} = 13.197$, $p < .001$, $\eta^2_p = 0.157$). Subsequent post-hoc tests revealed that there was no significant difference in the Simon effect in Go trials between electrode P7 and P8 ($t_{(72)} = 1.382$, $p = .171$). In contrast, the post-hoc analysis for NoGo trials revealed a significant difference in the magnitude of the observed Simon effect ($t_{(72)} = -3.174$, $p = .002$); with a significantly larger/more negative N1 Simon NoGo effect at electrode P7 (-0.223 µV ± 0.098) than at electrode P8 (0.170 µV ± 0.101). The remaining main and interaction effects that did not involve the group factor were not significant (all $F \leq 2.317$; all $p \geq .132$).

P2. Results for the P2 amplitude showed a main effect of condition ($F_{(1,73)} = 13.727$, $p < .001$, $\eta^2_p = 0.158$), with higher amplitudes in NoGo trials (2.131 µV ± 0.201) as compared to Go trials (1.858 µV ± 0.173). This result was confirmed with an add-on Wilcoxon signed rank test ($Z = -3.163$; $p = .002$). There was also a main effect of electrode ($F_{(1,73)} = 7.767$, $p = .007$, $\eta^2_p = 0.096$), showing overall larger P2 amplitudes at electrode P8 (2.293 µV ± 0.195) than at electrode P7 (1.695 µV ± 0.229). An additional Wilcoxon signed-rank test also confirmed this result ($Z = -2.418$; $p = .016$). Moreover, there was a three-way interaction of condition x congruency x electrode ($F_{(1,73)} = 6.096$, $p = .016$, $\eta^2_p = 0.077$). Post-hoc tests revealed significant differences in the magnitude of the P2 Simon effect in Go (-0.117 µV ± 0.054) and NoGo (0.099 µV ± 0.086) trials at electrode P8 ($t_{(74)} = 2.328$, p=.023). An add-on Wilcoxon signed rank test confirmed the statistically significant difference ($Z = -2.139$; $p = 0.032$). At electrode P7 however, no difference between the P2 Simon effect in Go and NoGo trials ($t_{(74)} = -0.465$,

p=0.643) was observed. The remaining main and interaction effects that did not involve the group factor were not significant (all F ≤ 3.729; all p ≥ .057).

N2. For the N2 amplitude, the analysis revealed a main effect of congruency ($F_{(1,73)}$ = 5.817, *p*=.018, η^2_p = 0.074), showing larger/more negative N2 amplitudes in incongruent trials (- 2.007 μV ± 0.205) than in congruent trials (-1.864 μV ± 0.192). There was also a main effect of electrode ($F_{(1,73)}$ =5.935, *p*= .017, η^2_p = 0.075), with larger amplitudes at electrode Cz (-2.096 μV ± 0.224) as compared to FCz (-1.775 μV ± 0.189). An additional Wilcoxon signed-rank tests confirmed both the main effect of congruency (*Z* = -2.345; *p* = .019) and electrode (*Z* = -2.181; *p* = .029). Furthermore, there was a significant interaction of condition x electrode ($F_{(1,73)}$ = 11.861, *p* < .001, η^2_p = 0.140). For Go trials, post-hoc tests show significantly more negative N2 amplitude at electrode Cz (-2.106 μV ± 0.252) as compared to FCz (-1.691 μV ± 0.203) ($t_{(74)}$ = -2.932, *p* = .004). Add-on non-parametric paired test confirmed the result (*Z* = -2.883; *p* = .004). For NoGo trials however, there was no significant difference in N2 amplitude between Cz and FCz ($t_{(74)}$ = -1.622, *p* = .109). There was also an interaction of congruency x electrode ($F_{(1,73)}$ = 5.298, *p*=.024, η^2_p = 0.068). Subsequent post-hoc tests for congruent trials indicated significantly larger N2 amplitudes at electrode Cz (-2.042 μV ± 0.242) as compared to FCz (-1.668 μV ± 0.193) ($t_{(74)}$ = -2.696, *p* = .009). This result was also confirmed with the Wilcoxon signed-rank test (*Z* = -2.572; *p* = .010). In contrast, there was no such difference for incongruent trials ($t_{(74)}$ = -1.911, *p* = .060). The remaining main and interaction effects that did not involve the group factor were not significant (all F ≤ 1.547; all p ≥ .218).

C-cluster

P3. The repeated measures ANOVA for the P3 amplitude showed a significant main effect of condition ($F_{(1,72)}$ = 179.852, *p* < .001, η^2_p = 0.714), with larger amplitudes than NoGo trials (4.617 μV ± 0.318) as compared to Go trials (1.616 μV ± 0.214). There was also a main effect of congruency ($F_{(1,72)}$ = 7.175, *p* = .009, η^2_p = 0.091), indicating higher amplitudes in incongruent (3.215 μV ± 0.259) than in congruent (3.019 μV ± 0.240) trials Furthermore, there was a main effect of electrode ($F_{(1,72)}$ = 92.090, *p* < .001, η^2_p = 0.561), with larger amplitudes at electrode Cz (3.745 μV ± 0.262) than at electrode FCz (2.489 μV ± 0.249). There was also an interaction of condition x electrode ($F_{(1,72)}$ = 12.641, *p* < .001, η^2_p = 0.149). Post-hoc tests were not run due to the interaction of group x condition x electrode detailed in the main manuscript. Additionally, there was a significant interaction of condition x congruency ($F_{(1,72)}$ = 13.448, *p* < .001, η^2_p = 0.157). Post-hoc tests were not run due to the interaction of group x condition x congruency detailed in the main manuscript. All other main and interaction effects that did not involve the group factor were not significant (all F ≤ 2.752; all p ≥ .101).

Figure S1: Scatterplots show the relationship between RIDE decomposed ERP signals and behavioral accuracy in NoGo trials, in the control group (left column) and reward group (right column). a) Relationships are shown for N1 Simon NoGo effect and mean NoGo accuracy (top row), P2 Simon NoGo effect and mean NoGo accuracy (bottom row). b) Relationships are shown for mean NoGo-N1 and Simon NoGo effect (top row), mean NoGo-P2 and Simon NoGo effect (bottom row), respectively. The fitted line (red) illustrates adjusted behavioral accuracy values as a function of adjusted decomposed ERP (predictor) values. Dashed-lines (blue) illustrates 95% confidence bounds of the fitted line.

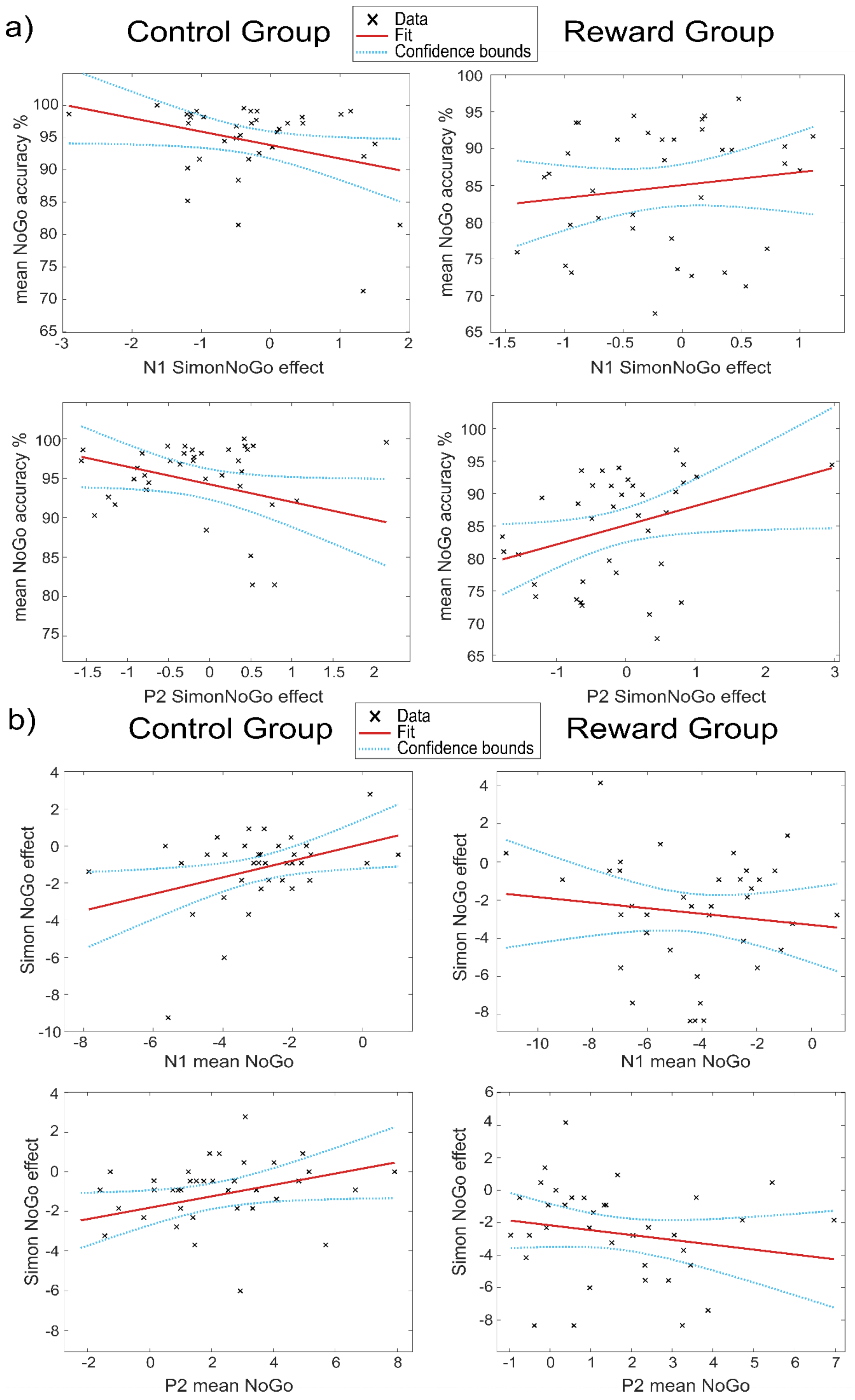
a)
Control Group
Data
Fit
Confidence bounds
Reward Group
mean NoGo accuracy %
N1 SimonNoGo effect
mean NoGo accuracy %
N1 SimonNoGo effect
mean NoGo accuracy %
P2 SimonNoGo effect
mean NoGo accuracy %
P2 SimonNoGo effect
b)
Control Group
Data
Fit
Confidence bounds
Reward Group
Simon NoGo effect
N1 mean NoGo
Simon NoGo effect
N1 mean NoGo
Simon NoGo effect
P2 mean NoGo
Simon NoGo effect
P2 mean NoGo

Neurophysiological Data: standard ERP data

Table 1. ERPs, corresponding electrodes and time windows (in milliseconds) in which the amplitudes were quantified as the average electrode activity.

**ERP**	**Electrode(s)**	**Time window (group)**	**Conditions**
N1	P7, P8	160 - 180 ms (both groups)	Go & NoGo condition
P2	P7, P8	260 - 295 ms (both groups)	Go & NoGo condition
N2	Cz, FCz	245 - 290 ms (reward group)<br>270-315 ms (control group)	Go & NoGo condition
P3	Cz, FCz	455-500 ms (both groups)	Go & NoGo condition

Exclusion criteria

Considering the standard ERP data, n=1 participant of the reward group was identified as extreme outliers in the N2 measure and therefore excluded from the analysis of the N2-ERP. For the N1, n= 2 participant from the control group were identified as extreme outliers and thus excluded from this particular analysis.

Event-related potential (ERP) results

The standard event-related potentials are shown in Figure S2.

Figure S2: Depicted are the standard ERPs. (a) NoGo conditions at electrode P7 in the N1 and P2 time window. (B) NoGo conditions at electrode Cz in the N2 and P3 time window. The time windows used for data quantification are provided in the Supplementary Table 1. The different lines show the congruent condition in control group (blue), the incongruent condition in control group (dotted blue line), the congruent condition in reward group (yellow) and the incongruent condition in reward group (dotted yellow line). The analyzed time windows were 20 ms around the peak of the C-cluster in the P3 time window in each condition as outlined in Supplementary Table 1.

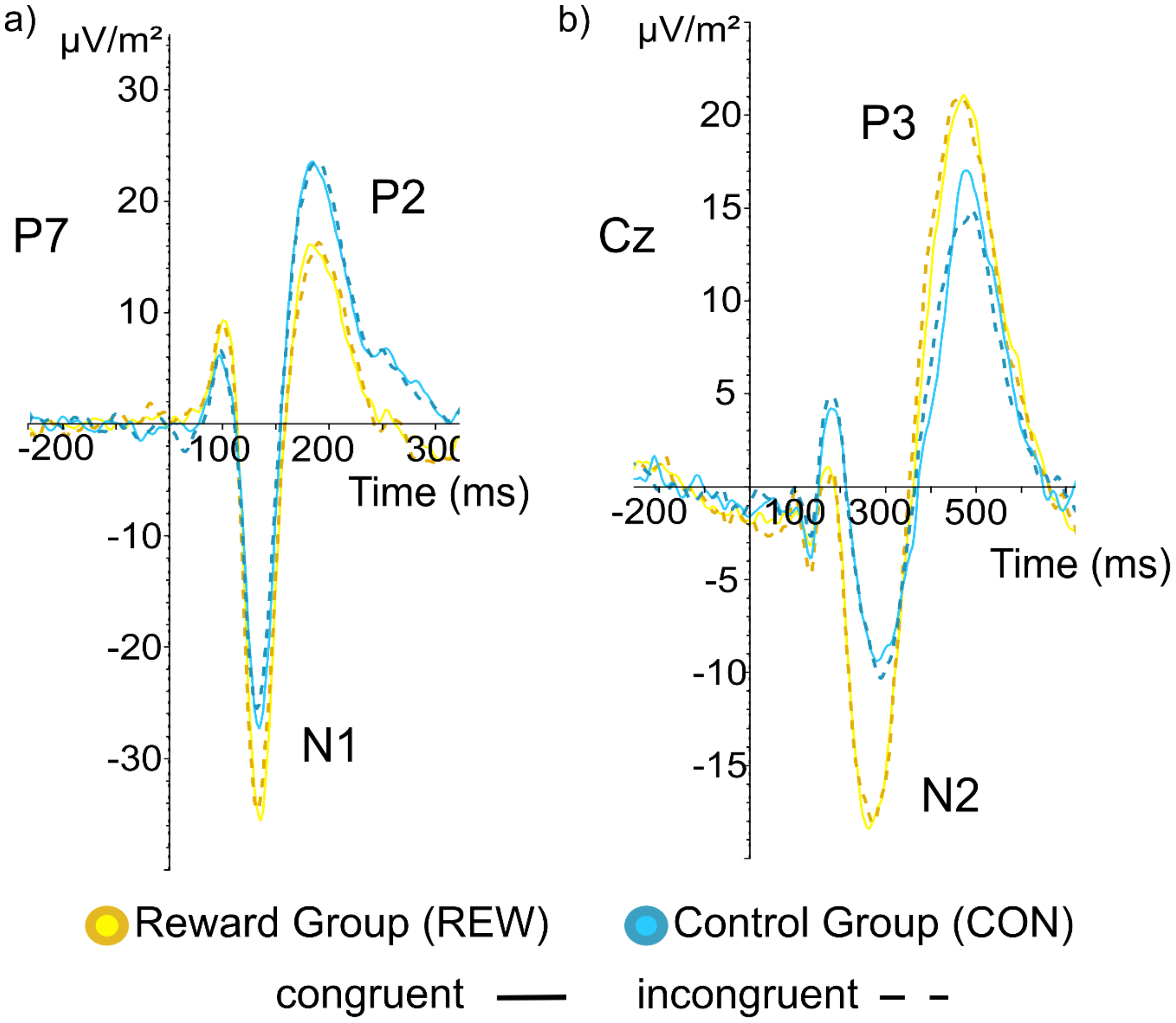

N1. The repeated measures ANOVA for the N1 component showed a main effect of group ($F_{(1,71)}$ = 10.348, p = .002, η^2_p = 0.127), indicating overall larger N1 amplitudes in the reward group (-34.523 µV/m^2 ± 2.515) as compared to the control group (-23.005 µV/m^2 ± 2.549). There was also an interaction of congruency x group ($F_{(1,71)}$ = 5.846, p = .018, η^2_p = 0.076) and in congruent trials, post hoc t-tests show larger N1 amplitudes in the reward group (-34.247 µV/m^2 ± 3.058) as compared to the control group (-23.338 µV/m^2 ± 1.828) ($t_{(58.628)}$ = 3.062; p = .003). For incongruent trials, we also found larger N1 amplitudes in the reward group (-34.800 µV/m^2 ± 3.078) as compared to the control group (-22.672 µV/m^2 ± 1.804) ($t_{(57.944)}$ = 3.400; p = .001). The interaction condition x group ($F_{(1,71)}$ = 2.562, p = .114, η^2_p = 0.035) did not reach significance, and an add-on Bayesian analysis provided anecdotal evidence for the H_0 (BF_{01} =

2.34). Finally, there was an interaction of congruency x electrode ($F_{(1,71)}$ = 7.539, p = .008, η^2_p = 0.096) and of condition x congruency x electrode ($F_{(1,71)}$ = 12.156, p < .001, η^2_p = 0.146). Post hoc analyses for the NoGo condition revealed an interaction of congruency x electrode ($F_{(1,72)}$ = 11.615; p = .001; η^2_p = 0.139). Further post-hoc t-tests for NoGo trials revealed a significant difference in the observed Simon effect ($t_{(72)}$ = -3.408, p = .001), with a smaller/more negative N1 Simon NoGo effect at electrode P7 (-1.531 µV/m^2 ± 0.654) than at electrode P8 (1.785 µV/m^2 ± 0.744). For Go trials however, no main or interaction effects were observed (all F ≤ 0.705; p ≥ .404). The remaining main and interaction effects that were not significant (all F ≤ 1.883; all p ≥ 0.174).

P2. The analysis for the P2 showed a main effect of condition ($F_{(1,73)}$ = 25.017, p < .001, η^2_p = 0.225), indicating overall larger P2 amplitudes in the NoGo condition (20.448 µV/m^2 ± 1.941) than in the Go condition (17.828 µV/m^2 ± 1.736). This result was confirmed by additional Wilcoxon signed-rank test (Z = -5.333, p < .001). There was also a main effect of electrode ($F_{(1,73)}$ = 4.091, p = .047, η^2_p = 0.053; P7: 17.371 µV/m^2 ± 2.088; P8: 20.905 µV/m^2 ± 1.591) as well as an interaction of electrode x group ($F_{(1,73)}$ = 4.948, p = .029, η^2_p = 0.063) showing that P2 amplitudes in the reward group were larger at electrode P8 (21.074 µV/m^2 ± 2.261) than at P7 (13.653 µV/m^2 ± 2.378) ($t_{(36)}$ = -2.776; p = .009). In the control no difference in P2 amplitudes between electrodes P7 and P8 were found ($t_{(37)}$ = 0.156; p = .877). Moreover, there were an interaction of electrode x congruency $F_{(1,73)}$ = 18.333, p < .001, η^2_p = 0.201 and an interaction of condition x congruency x electrode ($F_{(1,73)}$ = 4.631, p = .035, η^2_p = 0.060). Post hoc t tests demonstrated that P2 amplitudes were larger at electrode P7 (0.750 µV/m^2 ± 0.340) than at P8 (-0.544 µV/m^2 ± 0.350) in Go trials ($t_{(74)}$ = 3.101; p = .003), but not in Nogo trials ($t_{(74)}$ = -0.632; p = .529). Yet, the interaction of group x condition x congruency x electrode did not reach significance ($F_{(1,73)}$ = 2.444, p = .122) . An add-on Bayesian analysis for the highest interaction provided anecdotal evidence for H_1 (BF_{01} = 0.043). The remaining main and interaction effects were not significant (all other F ≤ 1.565; all p ≥ .215).

N2. For the N2 component, the ANOVA revealed a main effect of group ($F_{(1,72)}$ = 8.897, p = .004, η^2_p = 0.110), showing overall larger amplitudes in the reward group (-12.756 µV/m^2 ± 1.631) than in the control group (-5.967 µV/m^2 ± 1.587). Furthermore, there was a significant main effect of condition ($F_{(1,72)}$ = 112.277, p < .001, η^2_p = 0.609), revealing more negative N2 amplitudes in NoGo trials (-11.485 µV/m^2 ± 1.176) than in Go trials (-7.238 µV ± 1.134). The analysis further revealed a main effect congruency ($F_{(1,72)}$ = 11.681, p = .001, η^2_p = 0.140), showing that the N2 was larger on incongruent (-9.811 µV/m^2 ± 1.165) than on congruent trials (-8.912 µV/m^2 ± 1.125). There was also a main effect of electrode ($F_{(1,72)}$ =9.784, p = .003, η^2_p = 0.120; Cz: -10.914 µV/m^2 ± 1.210; FCz: -7.810 µV/m^2 ± 1.272), as well as an interaction of congruency x electrode ($F_{(1,73)}$ = 5.968, p = .017, η^2_p = 0.077). Add-on post hoc paired t-tests showed larger N2 amplitudes at electrode Cz than at FCz for both congruent trials ($t_{(73)}$ = -3.351; p = .001; Cz: -10.519 µV/m^2 ± 1.251; FCz: -7.115 µV/m^2 ± 1.337) and incongruent trials ($t_{(73)}$ = -2.900; p = .005; Cz: -11.128 µV/m^2 ± 1.287; FCz: -8.317 µV/m^2 ± 1.334)No interactions with the factor group were found, and all remaining interaction effects were not significant (all F ≤ 1.384; all p ≥ .243).

P3. For the P3 amplitude the analysis revealed a main effect of group ($F_{(1,73)}$ = 9.858, p = .002, η^2_p = 0.119), indicating overall larger amplitudes in the reward group (12.221 µV/m^2 ± 1.441) as compared to the control group (5.867 µV/m^2 ± 1.421). There was also a main effect of condition ($F_{(1,73)}$ =174.244, p < .001., η^2_p = 0.705), showing larger P3 amplitudes in NoGo (16.973 µV/m^2 ± 1.402) than in Go trials (1.116 µV/m^2 ± 0.896). Moreover, there was an

interaction of condition x congruency ($F_{(1,73)} = 5.528$, $p = .021$, $\eta^2_p = 0.070$) and an interaction of condition x congruency x group ($F_{(1,73)} = 7.268$, $p = .009$, $\eta^2_p = 0.091$). Post-hoc tests indicate a larger P3-Simon effect in Go trials in the reward group (0.126 µV/m² ± 0.645) than in the control group (-1.754 µV/m² ± 0.465) ($t_{(73)} = -2.375$; $p = 0.020$). The independent samples t-test for the P3-Simon NoGo effect did not reveal a difference between the groups ($t_{(73)} = 1.912$; $p = 0.060$). The ANOVA further revealed a main effect of electrode ($F_{(1,73)} = 11.875$, $p < .001$, $\eta^2_p = 0.140$), an interaction of electrode x condition ($F_{(1,73)} = 4.187$, $p = .044$, $\eta^2_p = 0.054$) and an interaction of electrode x condition x group ($F_{(1,73)} = 11.083$, $p = .001$, $\eta^2_p = 0.132$). Add on post hoc tests revealed group differences at both electrodes, that is, at electrode FCz significantly larger NoGo-P3 amplitudes were found in the reward group (21.508 µV/m² ± 2.359) as compared to the control group (10.129 µV/m² ± 1.882) ($t_{(73)} = -3.781$; $p < .001$). For the electrode Cz larger Go-P3 amplitudes were found in the reward group (5.556 µV/m² ± 1.731) than in the control group (0.979 µV/m² ± 1.149) ($t_{(73)} = -2.214$; $p = .030$), no differences were found in the NoGo-P3 at electrode Cz and for the Go-P3 at electrode FCz (all $t \leq |-1.995|$; $p \geq .054$). Lastly, there was an interaction of congruency x electrode ($F_{(1,73)} = 12.849$, $p < .001$, $\eta^2_p = 0.150$). In line with the other results post hoc t-test show significantly larger P3 amplitudes at electrode Cz than at FCz in both congruent trials ($t_{(74)} = 4.053$; $p < .001$; Cz: 10.881 µV/m² ± 1.188; FCz: 7.111 µV/m² ± 1.179) and incongruent trials ($t_{(74)} = 2.841$ $p = .006$; Cz: 10.444 µV/m² ± 1.158; FCz: 7.570 µV/m² ± 1.181). The remaining main and interaction effects were not significant (all $F \leq 3.197$; all $p \geq 0.078$).

Figure S3: Box plots illustrating the mean accuracy in percent for NoGo and Go condition, for the interaction group x condition. For the NoGo condition (left side), accuracy refers to correct, whereas in the Go condition (right side), accuracy was measured in correct responses. Horizontal line and x visualize the median and mean, respectively. The asterisk (*) indicates significant differences at $p < .05$.

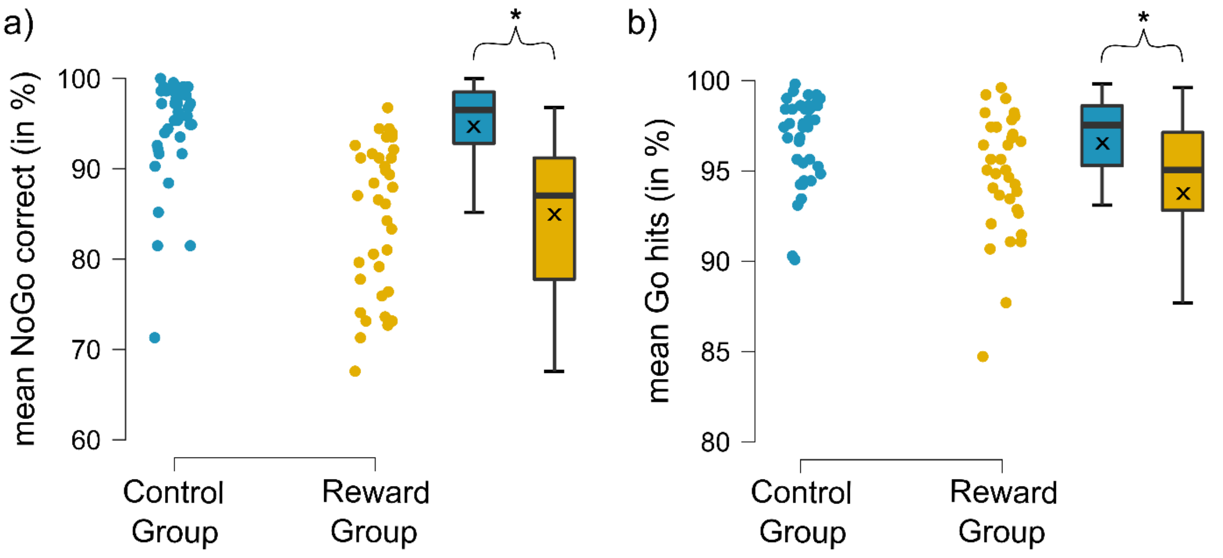

Supplement: Supplementary file 1 — Supplementary Information. [file 41598_2023_37524_MOESM1_ESM.pdf]
